# Plasma ADMA associates with all-cause mortality in renal transplant recipients

**DOI:** 10.1007/s00726-015-2023-0

**Published:** 2015-06-16

**Authors:** Anne-Roos S. Frenay, Else van den Berg, Martin H. de Borst, Bibiana Beckmann, Dimitrios Tsikas, Martin Feelisch, Gerjan Navis, Stephan J. L. Bakker, Harry van Goor

**Affiliations:** Department of Pathology and Medical Biology, University Medical Center Groningen and University of Groningen, Hanzeplein 1, 9700 RB Groningen, The Netherlands; Nephrology, University Medical Center Groningen and University of Groningen, Groningen, The Netherlands; Centre of Pharmacology and Toxicology, Hannover Medical School, Hannover, Germany; Clinical and Experimental Sciences, Faculty of Medicine, Southampton General Hospital, University of Southampton, Southampton, UK

**Keywords:** Asymmetric dimethylarginine, Kidney, Survival, Transplantation

## Abstract

Asymmetric dimethylarginine (ADMA) is a key endogenous inhibitor of endothelial NO synthase that affects endothelial function, blood pressure and vascular remodeling. Increased plasma levels of ADMA are associated with worse outcome from cardiovascular disease. Due to endothelial dysfunction before and after kidney transplantation, renal transplant recipients (RTR) are at high risk for the alleged deleterious effects of ADMA. We investigated the associations of ADMA levels with all-cause mortality and graft failure in RTR. Plasma ADMA levels were determined in 686 stable outpatient RTR (57 % male, 53 ± 13 years), with a functioning graft for ≥1 year. Determinants of ADMA were evaluated with multivariate linear regression models. Associations between ADMA and mortality were assessed using multivariable Cox regression analyses. The strongest associations with plasma ADMA in the multivariable analyses were male gender, donor age, parathyroid hormone, NT-pro-BNP and use of calcium supplements. During a median follow-up of 3.1 [2.7–3.9] years, 79 (12 %) patients died and 45 (7 %) patients developed graft failure. ADMA was associated with increased all-cause mortality [HR 1.52 (95 % CI 1.26–1.83] per SD increase, *P* < 0.001], whereby associations remained upon adjustment for confounders. ADMA was associated with graft failure [HR 1.41 (1.08–1.83) per SD increase, *P* = 0.01]; however, upon addition of eGFR significance was lost. High levels of plasma ADMA are associated with increased mortality in RTR. Our findings connect disturbed NO metabolism with patient survival after kidney transplantation.

## Introduction

The increased incidence of chronic kidney disease (CKD) over the last decades is related to the aging population and to lifestyle-related diseases, e.g., hypertension, atherosclerosis and diabetes. For patients progressing to end-stage renal disease, renal replacement therapy is the final treatment option. When compared to the general population, patients receiving renal replacement therapy are at increased risk of infections, malignancies and cardiovascular events (Vogelzang et al. 2015). In renal transplant recipients (RTR), one of the main pathophysiological processes that contribute to premature cardiovascular disease is endothelial dysfunction. Different processes including decreased expression of glomerular endothelial nitric oxide synthase (eNOS) expression after renal transplantation (Albrecht et al. 2002) are thought to play a role in these adverse events. The subsequent reduction in NO release may cause vascular damage through changes in the renal hemodynamics (Nakayama et al. 2009) and enhanced endothelial adhesion of leukocytes and platelets (Huang et al. 1995).

Asymmetric dimethylarginine (ADMA) is an endogenous inhibitor of endothelial nitric oxide (NO) synthase and thereby considered an adverse mediator of endothelial function (Cooke 2000; Yilmaz et al. 2006). ADMA is associated with cardiovascular risk factors in patients with hypertension (Surdacki et al. 1999), diabetes (Can et al. 2011) and hyperlipidemia (Boger et al. 1998). This is further evidenced by increased plasma levels of ADMA in patients with CKD, which are linked to both the development and the progression of CKD (Ravani et al. 2005; Fliser et al. 2005; Hanai et al. 2009). In pre-dialysis patients with CKD, circulating levels of ADMA are an independent risk factor for left ventricular hypertrophy with predictive value for cardiovascular events (Shi et al. 2010). Interestingly, a recent study in patients with CKD demonstrated a link between levels of ADMA and fibroblast growth factor-23 (FGF-23), which by itself is also linked to markers of endothelial cell injury (Malyszko et al. 2014), in the development of endothelial dysfunction (Yilmaz et al. 2010). Furthermore, a recent study in CKD patients suggested that the association between ADMA level and CKD progression is modified by FGF23 (Tripepi et al. 2015). Whether this effect modification also occurs in renal transplant recipients for graft and patient survival, or whether the association between ADMA and these outcomes is mediated by FGF-23 is unknown.

Based on the characteristics of ADMA, especially those related to endothelial dysfunction, we hypothesized that ADMA is associated with graft failure and mortality after kidney transplantation. We investigated this in a cohort of 686 renal transplant recipients with long-term follow-up.

## Methods

### Study design and population

From November 2008 till June 2011, all stable RTR (≥18 years, *n* = 817) with a functioning graft for over 1 year that visited the outpatient clinic of the University Medical Center Groningen (UMCG), the Netherlands were invited to participate. After giving written informed consent, a total of 707 (87 %) RTR participated in the present study. Plasma ADMA was measured in samples of 686 RTR (97 %). Further details of the study population have been published previously (van den Berg et al. 2012a, b, 2014). The study protocol was approved by the Review Board of the UMCG (METc 2008/186) and was in adherence to the Declaration of Helsinki.

### Outcome parameters

The primary outcome measures of this study were death-censored graft failure (defined as restart of dialysis or re-transplantation) and all-cause mortality. Outcome measures were recorded until the end of May 2013, with no participants lost to follow-up.

### Clinical parameters

As described previously (van den Berg et al. 2014), all participants were instructed to collect 24-h urine sample at the day prior to their visit to the outpatient clinic. First, blood pressure and heart rate were measured using a semi-automatic device (Dinamap^®^ 1846, Critikon, Tampa, FL, USA) every minute for the duration of 15 min, following a strict protocol (van den Berg et al. 2012a, b). An average of the last three values was taken as a final value. Body weight and height were measured and body mass index (BMI) was calculated as weight divided by height square (kg/m^2^), body surface area was calculated using the universally adopted formula of DuBois & DuBois (Dubois and Dubois 1989). In the morning after an overnight fasting period, blood was drawn and subsequently venous blood gas analyses were performed photometrically. Electrolytes, phosphate, albumin, urea and creatinine in plasma and urine were measured using routine laboratory methods, which was also the case for serum cholesterol, HbA1c and hsCRP. Renal function was assessed by calculating the estimated glomerular filtration rate (eGFR) using the CKD Epidemiology Collaboration (CKD-EPI) equation (Levey Levey et al. 2009). Serum calcium was corrected for hypoalbuminemia (<40 g/L) using the following formula: corrected calcium = serum calcium (mmol/L) + 0.02 × [40 − serum albumin (g/L)]. Intact FGF-23 was measured using a commercially available ELISA kit (Kainos Laboratories, Inc., Tokyo, Japan) (Baia et al. 2014). Information on participants’ health status, medical history and medication use was extracted from patient records. Relevant transplant information was extracted from the UMCG renal transplant database. Smoking behavior was categorized to current, former or never smoked, using a self-report questionnaire.

### Plasma ADMA

Free ADMA was measured by a previously described fully validated GC–MS/MS method (Tsikas et al. 2003). The analyses were performed on a ThermoQuest TSQ 7000 mass spectrometer (Finnigan MAT, San Jose, CA, USA). ADMA was quantified by selected-reaction monitoring (SRM) of the transitions *mlz* 634 → *mlz* 378 for endogenous ADMA, whereas for the internal standard *mlz* 637 → *mlz* 378 was used. The dwell time was 100 ms for each analyte and each transition. The basal plasma concentration of the QC samples was 380 nM for ADMA.

### Statistical analysis

Statistical analyses were performed using SPSS 22.0 for Windows (SPSS Corp. Chicago, IL, USA) and GraphPad Prism version 5.00 for Windows (GraphPad Software, San Diego, CA, USA). Non-normally distributed parameters were presented as median [interquartile range (IQR)] and normally distributed variables were expressed as mean ± standard deviation (SD). A two-sided *P* value <0.05 was considered statistically significant. Histograms and probability plots were displayed followed by the Kolmogorov–Smirnov test to test the distribution of all parameters. When skewed, parameters were normalized for analyses by logarithmic transformation [high-sensitive C-reactive protein (CRP), triglycerides, albuminuria, FGF-23, N-terminal pro-brain natriuretic peptide (NT-pro-BNP), parathyroid hormone (PTH)]. The study population was subdivided into tertiles of ADMA to visualize potential associations of plasma ADMA with different parameters in RTR. To establish *P* values for differences in ADMA tertiles, an ANOVA was used for normally distributed continuous data, whereas the Kruskal–Wallis test was used for non-normally distributed data and the χ^2^-test for nominal data. To identify the independent determinants of ADMA, univariable and multivariable linear regression analyses were performed. Multivariable linear regression models were constructed using backward selection (*P*_out_ > 0.05), which included all twenty-one variables that were significantly associated with ADMA in the univariable analysis. Tertiles of ADMA were tested for associations with all-cause mortality and death-censored graft failure by Kaplan–Meier analysis, including the log-rank test. For the Cox regression analyses, models were constructed with inclusion of the potential confounders of ADMA. These are the parameters that significantly associated with plasma ADMA in the multivariable analysis. We first performed crude Cox regression analyses (model 1) and analyses with adjustment for age and gender (model 2). In addition, eGFR was added (model 3), and we adjusted for the potential confounders of ADMA, which were identified in the multivariable regression analysis (donor age, serum PTH, NT-pro-BNP, use of calcium supplements) (model 4). In the final model, we included intact FGF-23, to test whether the association of ADMA with all-cause mortality was influenced by this parameter (model 5). The same models were used to test the association of plasma ADMA with graft failure. We explored a potential interaction by FGF-23 for the association between ADMA and mortality in the full Cox regression model by adding the interaction term ADMA × FGF-23.

## Results

### Patient characteristics according to tertiles of plasma ADMA

**Fig. 1:**
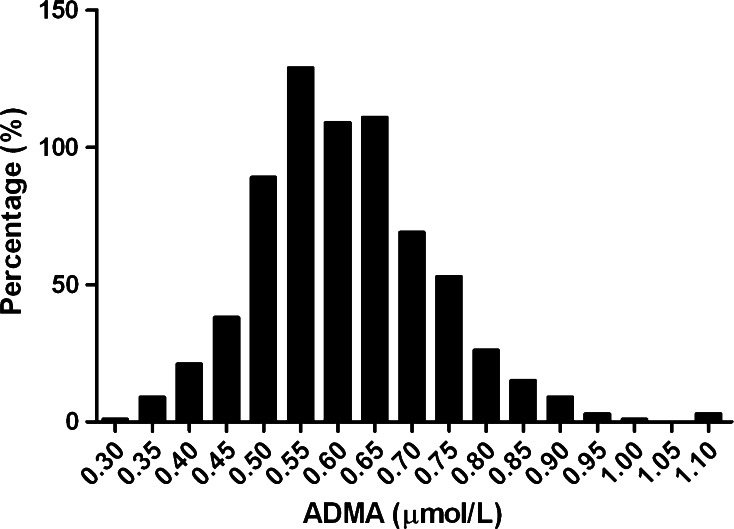
Histogram of plasma ADMA showing normal distribution curve in RTR. Plasma ADMA (0.61 ± 0.12 µmol/L) measured in 686 renal transplant recipients was normally distributed

Plasma ADMA was normally distributed and had a mean value of 0.61 ± 0.12 µmol/L (Fig. 1). The patient cohort of 686 RTR had a mean age of 53.0 ± 12.7 years and 57 % were male. Baseline characteristics of the patient cohort per tertile of ADMA are displayed in Table 1. Median time between renal transplantation and baseline measurement was 5.4 [1.9–12.0] years. RTR in the highest tertile of ADMA were older and more likely to be male compared to RTR in the other tertiles, whereas the other demographic parameters were similar among ADMA tertiles. With regard to transplant characteristics, RTR with the highest ADMA levels less often received their kidney from living donors, and their donors tended to be older.

**Table 1:** Baseline patient characteristics presented as tertiles of plasma ADMA

	Renal transplant recipients tertiles of ADMA				
	Overall (*N* = 686)	Tertile 1 (*N* = 249)	Tertile 2 (*N* = 213)	Tertile 3 (*N* = 224)	*P* value
ADMA (µmol/L)	0.61 ± 0.12	≤0.56	0.57–0.65	≥0.66	<0.001
Demographics					
Age, years	53 ± 13	50 ± 13	54 ± 12	55 ± 13	**<0.001**
Male gender	390 (57)	127 (51)	125 (59)	138 (62)	**0.05**
Current smoker, *n* (%)	82 (13)	29 (13)	31 (15)	22 (11)	0.38
Current diabetes, *n* (%)	165 (24)	51 (21)	51 (24)	63 (28)	0.15
BMI, kg/m^2^	27 ± 5	27 ± 5	27 ± 5	27 ± 5	0.84
BSA, m^2^	1.94 ± 0.22	1.94 ± 0.19	1.95 ± 0.22	1.94 ± 0.22	0.72
Systolic blood pressure, mmHg	136 ± 17	136 ± 16	135 ± 18	138 ± 15	0.26
Diastolic blood pressure, mmHg	83 ± 11	83 ± 10	82 ± 12	82 ± 11	0.44
Heart rate, bpm	69 ± 12	69 ± 13	68 ± 12	69 ± 11	0.61
Renal transplantation					
Transplant vintage, years	5.4 [1.9–12.1]	5.2 [2.2–10.8]	5.2 [2.0–11.6]	6.1 [1.6–14.0]	0.51
Living donor, *n* (%)	229 (34)	103 (42)	70 (34)	56 (26)	**0.001**
Pre-emptive KTx, *n* (%)	112 (16)	51 (21)	33 (16)	28 (13)	0.06
HLA mismatches, *n*	2 [1–3]	2 [1–3]	2 [1–3]	2 [1–3]	0.31
Age donor, years	43 ± 16	40 ± 16	43 ± 15	45 ± 15	**0.009**
Acute rejection, *n* (%)	181 (26)	65 (26)	54 (26)	62 (28)	0.86
Laboratory measurements					
Hemoglobin, mmol/L	8.2 ± 1.1	8.3 ± 1.0	8.2 ± 1.1	8.1 ± 1.2	0.09
HbA1C, %	6.0 ± 0.8	5.9 ± 0.8	6.0 ± 0.8	6.0 ± 0.9	0.59
eGFR, CKD-EPI (ml/min/1.73 m^2^)	52.2 ± 20.2	57.9 ± 21.4	51.2 ± 18.4	46.9 ± 19.0	**<0.001**
Corrected calcium mmol/L	2.34 ± 0.15	2.33 ± 0.15	2.35 ± 0.14	2.35 ± 0.14	0.42
Phosphate, mmol/L	0.97 ± 0.21	0.94 ± 0.21	0.96 ± 0.21	1.01 ± 0.21	**0.002**
Magnesium, mmol/L	0.95 ± 0.12	0.95 ± 0.12	0.95 ± 0.13	0.96 ± 0.12	0.81
PTH, pmol/L	8.9 [5.9–14.7]	8.1 [5.6–12.0]	8.7 [6.2–15.4]	11.0 [6.5–17.3]	**0.001**
Venous pH	7.37 ± 0.04	7.37 ± 0.04	7.37 ± 0.04	7.36 ± 0.04	**0.009**
Venous HCO_3_^−^, mmol/L	24.6 ± 3.1	24.8 ± 2.9	24.8 ± 3.2	24.2 ± 3.2	0.07
hsCRP, mg/L	1.6 [0.7–4.5]	1.6 [0.7–4.6]	1.8 [0.6–5.0]	1.5 [0.8–4.4]	0.91
Albumin, g/L	43.0 ± 3.0	43.6 ± 2.8	42.9 ± 2.8	42.4 ± 3.2	**<0.001**
Alkaline phosphatase, U/L	67 [54–83]	66 [51–79]	67 [56–82]	69 [54–92]	0.12
FGF-23, pg/mL	61 [43–99]	54 [39–82]	60 [46–93]	75 [53–126]	**<0.001**
Total cholesterol, mmol/L	5.0 [4.4–5.8]	5.1 [4.4–5.8]	5.0 [4.4–5.8]	5.1 [4.2–5.9]	0.95
HDL cholesterol, mmol/L	1.3 [1.1–1.6]	1.4 [1.1–1.7]	1.3 [1.1–1.7]	1.3 [1.0–1.5]	**0.002**
LDL cholesterol, mmol/L	2.9 [2.3–3.5]	2.9 [2.4–3.5]	2.9 [2.2–3.5]	2.9 [2.3–3.6]	0.83
Triglycerides, mmol/L	1.68 [1.25–2.30]	1.63 [1.13–2.23]	1.73 [1.29–2.43]	1.69 [1.28–2.3]	0.17
NT-pro-BNP, ng/L	252 [108–634	150 [76–405]	229 [109–565]	396 [185–1086]	**<0.001**
Albuminuria, mg/24 h	40 [11–177]	29 [8–154]	28 [10–103]	83 [13–300]	**0.001**
Medication					
Anti-hypertensives, *n* (%)	606 (88)	212 (85)	190 (89)	204 (91)	0.12
Statins, *n* (%)	361 (53)	132 (53)	105 (50)	361 (53)	0.45
Calcium supplements, *n* (%)	147 (21)	46 (19)	46 (22)	55 (25)	0.27
Vitamin D supplements	168 (25)	63 (25)	44 (21)	61 (27)	0.26
Vitamin K antagonists	77 (11)	19 (8)	22 (10)	36 (16)	**0.01**
Prednisone, mg/d	10 [7.5–10]	10 [7.5–10]	10 [7.5–10]	10 [7.5–10]	0.19
Calcineurin inhibitors	391 (57)	120 (48)	134 (63)	137 (61)	**0.002**
Proliferation inhibitor	572 (83)	222 (89)	171 (80)	179 (80)	**0.009**
Sirolimus	13 (2)	5 (2)	7 (3)	1 (1)	0.10

Data are presented as mean ± SD, number (percentage) or median (IQR). Statistical analysis was performed using ANOVA, Kruskal–Wallis or χ^2^-test when appropriate. Bold indicates statistical significance (*P* < 0.05)

*ADMA* asymmetrical dimethylarginine, *BSA* body surface area, *eGFR* estimated glomerular filtration rate, *HbA1c* glycated hemoglobin, *HCO*_*3*_^*−*^ bicarbonate, *HDL* high-density lipoprotein, *HLA* human leukocyte antigen, *hsCRP* high-sensitivity C-reactive protein, *KTx* kidney transplantation, *LDL* low-density lipoprotein, *PTH* parathyroid hormone

Estimated GFR was lower and urinary protein excretion higher in RTR with the highest ADMA. Serum phosphate, PTH and levels of NT-pro-BNP were significantly increased in RTR in the highest ADMA tertile, whereas serum albumin levels, HDL cholesterol and venous pH were significantly lower when compared to RTR with the lowest ADMA level. RTR with the highest levels of ADMA also had the highest levels of intact FGF-23. With regard to medication use, RTR with the highest plasma ADMA more often used vitamin K antagonist and calcineurin inhibitors when compared to the lowest ADMA tertile, while the use of proliferation inhibitors was less. Table 2 provides an overview of associations of ADMA levels with different parameters in univariable and multivariable regression analyses. The strongest associations with plasma ADMA in the multivariable analyses were male gender, donor age, PTH, NT-pro-BNP and use of calcium supplements. When adding NT-pro-BNP to the linear regression analysis with backward selection, eGFR lost its significant association with ADMA.

**Table 2:** Associations of plasma ADMA with clinical parameters in RTR

	Plasma ADMA			
	Univariable		Multivariable	
	St. Beta	*P* value	St. Beta	*P* value
Demographics				
Age, years	0.123	**<0.001**		
Male gender	0.082	**0.03**	0.133	**0.001**
Current smoker	−0.011	0.78		
Current diabetes	0.071	0.06		
BMI, kg/m^2^	−0.046	0.23		
BSA, m^2^	−0.046	0.23		
SBP, mmHg	0.026	0.50		
DBP, mmHg	−0.039	0.31		
Heart rate, bpm	0.006	0.87		
Renal transplantation				
Transplant vintage, years	0.04	0.30		
Living donor	−0.127	**0.001**		
Pre-emptive KTx	−0.098	**0.01**		
HLA mismatches	−0.022	0.57		
Age donor, years	0.110	**0.005**	0.094	**0.02**
Acute rejection	0.016	0.67		
Laboratory measurements				
Hemoglobin, mmol/L	−0.082	**0.03**		
HbA1C, %	0.010	0.80		
eGFR, CKD-EPI (ml/min/1.73 m^2^)	−0.209	**<0.001**		
Corrected calcium mmol/L	0.059	0.13		
Phosphate, mmol/L	0.107	**0.005**		
Magnesium, mmol/L	0.014	0.72		
PTH, pmol/L	0.119	**0.002**	0.104	**0.01**
Venous pH	−0.123	**0.002**		
Venous HCO_3_, mmol/L	−0.108	**0.006**		
hsCRP, mg/L	−0.016	0.68		
Albumin, g/L	−0.160	**<0.001**		
Alkaline phosphatase, U/L	0.058	0.13		
FGF-23, pg/mL	0.185	**<0.001**		
Total cholesterol, mmol/L	−0.052	0.18		
HDL cholesterol, mmol/L	−0.139	**<0.001**		
LDL cholesterol, mmol/L	−0.021	0.58		
Triglycerides, mmol/L	0.036	0.34		
NT-pro-BNP, ng/L	0.271	**<0.001**	0.265	**<0.001**
Albuminuria, mg/24 h	0.115	**0.003**		
Medication				
Anti-hypertensives	0.081	**0.03**		
Statins	0.032	0.40		
Calcium supplements	0.085	**0.03**	0.088	**0.03**
Vitamin D supplements	0.029	0.44		
Vitamin K antagonists	0.125	**0.001**		
Prednisone, mg/d	0.055	0.15		
Calcineurin inhibitors	0.092	**0.02**		
Proliferation inhibitor	−0.101	**0.008**		
Sirolimus	−0.065	0.10		

Regression coefficients are given as standardized betas, i.e., change of cardiovascular parameter in SD, per SD increase of plasma ADMA level

*P* values less than 0.05 are in bold

*ADMA* asymmetrical dimethylarginine, *BMI* body mass index, *BSA* body surface area, *SBP* systolic blood pressure, *DBP* diastolic blood pressure, *FGF-23* fibroblast growth factor 23, *hsCRP* high-sensitive C-Reactive Protein, *HDL cholesterol* high-density lipoprotein, *LDL* low-density lipoprotein, *NT-pro-BNP* N-terminal pro-Brain Natriuretic peptide, PTH parathyroid hormone, *HbA1c* glycated hemoglobin, *eGFR* estimated glomerular filtration rate

### ADMA is associated with increased all-cause mortality in RTR, independent of FGF-23

**Fig. 2:**
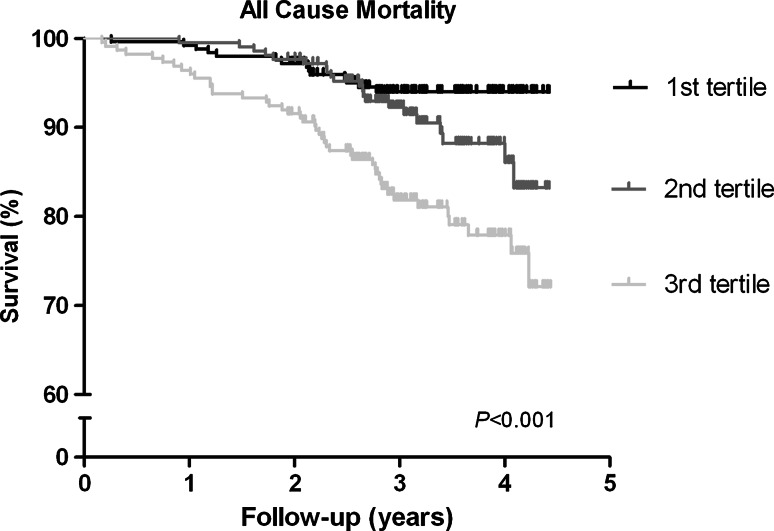
Kaplan–Meier plot of the association of ADMA with all-cause mortality in RTR. Higher plasma levels of ADMA are associated with significantly increased all-cause mortality in renal transplant recipients. Kaplan–Meier curve displayed for all-cause mortality, with log-rank test *P* value <0.001

Of the 686 RTR in our cohort, 79 (12 %) died within a median follow-up period of 3.1 [2.7–3.9] years. In the highest tertile of ADMA, 44 out of 224 (20 %) died, while this was 22 out of 213 (10 %) in the middle tertile and 13 out of 249 (5 %) in the tertile with the lowest ADMA levels (log-rank test *P* < 0.001, Fig. 2). In addition, we performed Cox regression analyses with potential confounders of plasma ADMA that were identified in the multivariable regression analyses (Table 3). The crude Cox regression analysis (model 1) showed that plasma ADMA is associated with increased mortality risk [HR 1.52 (95 % CI 1.26–1.83), *P* < 0.001 per SD increase]. After adjusting for age and gender [model 2; HR 1.52 (1.22–1.88), *P* < 0.001], for eGFR [model 3; HR 1.43 (1.15–1.78), *P* = 0.001] and other potential confounders in multivariate regression analysis [model 4; HR 1.34 (1.07–1.68), *P* = 0.01], plasma ADMA remained significantly associated with increased mortality risk in RTR. In the final model, we also added FGF-23, however, this did not affect the association of plasma ADMA with all-cause mortality [model 5; HR 1.34 (1.07–1.68), *P* = 0.01]. We found no significant interaction by FGF-23 for the association between ADMA and mortality (*P* = 0.23).

**Table 3:** Associations of plasma ADMA with all-cause mortality in RTR

	Plasma ADMA (continuous)	
	HR (95 % CI) per SD	*P* value
Model 1	1.52 (1.26–1.83)	**<0.001**
Model 2	1.52 (1.22–1.88)	**<0.001**
Model 3	1.43 (1.15–1.78)	**0.001**
Model 4	1.34 (1.07–1.68)	**0.01**
Model 5	1.34 (1.07–1.68)	**0.01**

Model 1: crude, Model 2: adjusted for age, gender, Model 3: as model 2, additionally adjusted for Egfr, Model 4: as model 3, additionally adjusted for donor age, PTH, NT-pro-BNP, use of calcium supplements, Model 5: as model 4, additionally adjusted for FGF-23

*P* values less than 0.05 are in bold

*ADMA* asymmetric dimethylarginine, *CI* confidence interval, *FGF-23* fibroblast growth factor 23, *HR* hazard ratio, *NT-pro-BNP* N-terminal pro-hormone of brain natriuretic peptide, *PTH* parathyroid hormone, *SD* standard deviation

### ADMA is associated with graft failure in RTR; lost significance after adding renal function

In our cohort, 45 (7 %) RTR developed graft failure in a median follow-up period of 3.1 [2.7–3.9] years. In the highest tertile of ADMA, 19 out of 224 (9 %) developed graft failure, while this was 16 out of 213 (8 %) and 10 out of 249 (4 %) in the middle and lowest tertile of ADMA, respectively (log-rank test *P* = 0.10). In the crude Cox regression analysis, plasma ADMA was significantly associated with graft failure [HR 1.41 (1.08–1.83), *P* = 0.01] (Table 4). Upon adjustment for age and gender, plasma ADMA remained significantly associated with graft failure [HR 1.42 (1.11–1.82), *P* = 0.01]. However, when adding eGFR, the significant association of ADMA with graft failure was lost (HR 1.26 (0.95–1.68), *P* = 0.11].

**Table 4:** Associations of plasma ADMA with graft failure in RTR

	Plasma ADMA (continuous)	
	HR (95 % CI) per SD	*P* value
Model 1	1.41 (1.08–1.83)	**0.01**
Model 2	1.42 (1.11–1.82)	**0.01**
Model 3	1.26 (0.95–1.68)	0.11
Model 4	1.11 (0.81–1.51)	0.52
Model 5	1.10 (0.80–1.49)	0.57

Model 1: crude, Model 2: adjusted for age, gender, Model 3: as model 2, additionally adjusted for eGFR, Model 4: as model 3, additionally adjusted for donor age, PTH, NT-pro-BNP, use of calcium supplements, Model 5: as model 4, additionally adjusted for FGF-23

*P* values less than 0.05 are in bold

*ADMA* asymmetric dimethylarginine, *CI* confidence interval, *FGF-23* fibroblast growth factor 23, *HR* hazard ratio, *NT-pro-BNP* N-terminal pro-hormone of brain natriuretic peptide, *PTH* parathyroid hormone, *SD* standard deviation

## Discussion

Plasma ADMA is associated with increased risk of all-cause mortality in stable renal transplant recipients. This association was solid and independent of various potential confounders. These results are in line with the current conception of ADMA as a serious risk factor for cardiovascular disease, the primary cause of death in RTR.

ADMA is an endogenous inhibitor of NO synthase that has the potential to negatively affect endothelial function, blood pressure and vascular remodeling (Leiper 2005) via reduction of the production of NO. High levels of ADMA are acknowledged as a risk factor for cardiovascular disease in CKD patients (Abedini et al. 2010; Lu et al. 2011; Ravani et al. 2005) and associate with increased mortality in patients undergoing coronary angiography (Meinitzer et al. 2011). Where others have described ADMA and NT-pro-BNP as independent risk markers (Duckelmann et al. 2007), the results of the present study demonstrate a strong association between plasma ADMA and NT-pro-BNP in RTR. Despite this strong association, our results show that ADMA still is an independent risk factor associated with mortality in RTR. Since ADMA significantly inhibits NOS and reduces NO production in vitro in endothelial cells and isolated human blood vessels (Faraci et al. 1995) and ADMA administration to healthy rats (Gardiner et al. 1993) as well as healthy humans (Kielstein et al. 2004) induced increased blood pressure, increased renal vasculature resistance and decreased cardiac output, one might hypothesize that high levels of ADMA can cause left ventricular wall stress with increased NT-pro-BNP levels. The link between endothelial dysfunction and vascular hypertrophy has already been demonstrated in end-stage renal disease patients (Zoccali et al. 2002). Furthermore, in patients with CKD, plasma ADMA was addressed as an independent risk factor for cardiac hypertrophy and associated with cardiovascular events (Shi et al. 2010).

We found a borderline significant inverse relationship between ADMA and renal function in RTR. This association was also demonstrated in CKD patients (Ravani et al. 2005; Fliser et al. 2005; Tripepi et al. 2015), whereby ADMA predicted the incidence rate of renal events (decrease in eGFR of >30 %, dialysis, or kidney transplantation (Tripepi et al. 2015). The association of ADMA with renal function decline and renal events might be explained by its inhibitory effects on NO production, which in turn leads to deteriorated renal function and renal fibrosis (Mihout et al. 2011). However, in the present study we demonstrated that, by the addition of NT-pro-BNP to the multivariate model, eGFR lost its significant association with ADMA. Apparently, NT-pro-BNP is an even stronger determinant of ADMA than renal function. This might be related to the fact that they are both involved in cardiovascular dynamics.

Furthermore, others found a link between levels of ADMA and graft failure (Abedini et al. 2010). In the present study, we confirmed this association; however, significance was lost after adjustment for renal function. This can be explained by the fact that renal function by itself is a strong predictor for graft failure (Hariharan et al. 2002) and might also be a consequence of the low number of events in our cohort. Thereby, in the present study, the way of classifying graft failure differed from Abedini et al., since we only took into account the RTR that returned to dialysis or had to undergo re-transplantation, whereas they also included doubling of serum creatinine into this category (Abedini et al. 2010). Our study is in line with the work of Abedini et al. who, in a cohort of renal transplant recipients with slightly different patients’ characteristics, also found a significant association between ADMA and mortality. However, our study adds that ADMA is significantly associated with NT-pro-BNP, but is independently associated with all-cause mortality. This latter also holds true for ADMA and FGF-23.

A potential shared causal pathway of FGF-23 and ADMA in the development of endothelial dysfunction was recently proposed (Yilmaz et al. 2010). In line with this, FGF-23 was previously reported to modify the association between ADMA and renal function loss in CKD patients (Tripepi et al. 2015). In the present study, we examined whether the association of plasma ADMA with mortality is mediated by FGF-23, however, from our results we conclude that the association between ADMA and all-cause mortality is independent of plasma intact FGF-23 in RTR. We also found no evidence of interaction by FGF-23 for the association between ADMA and mortality in our cohort.

One of the strengths of our study is the large sample size of well-defined, stable RTR. Extensive data collection, including data from 24-h urine samples allowed for adjustment for many confounders. Despite this, our study is strictly an observational epidemiological study. Causality is, therefore, hard to prove. Since little is known about how ADMA affects cardiovascular parameters in this cohort, other factors might underlie the observed associations. Furthermore, our study population consisted predominantly of Caucasian people, which calls prudence to extrapolation of our results to populations of other ethnicities. Furthermore, we need to keep in mind that circulating levels of ADMA in some cases reflect the intracellular concentrations (Davids et al. 2012), however, they are not necessarily in equilibrium with each other (Davids and Teerlink 2013).

In conclusion, high levels of plasma ADMA are associated with increased mortality in RTR. Since it is not yet clear whether ADMA is rather a progression marker of disease, a novel risk factor of disease or both, additional studies to sort out these issues are warranted. Currently, therapies to reduce ADMA levels are being tested for their efficacy, as well as to evaluate their therapeutic effect in diseases characterized by endothelial dysfunction.

## Abbreviations

ADMA: Asymmetrical dimethylarginine
BMI: Body mass index
BSA: Body surface area
CKD: Chronic kidney disease
DBP: Diastolic blood pressure
DDAH: Dimethylarginine dimethylaminohydrolase
Egfr: Estimated glomerular filtration rate
FGF-23: Fibroblast growth factor 23
HbA_1c_: Glycated hemoglobin
hsCRP: High-sensitive C-reactive protein
HDL: High-density lipoprotein
HLA: Human leukocyte antigen
HR: Hazard risk
IQR: Interquartile range
KTx: Kidney transplantation
LDL: Low-density lipoprotein
eNOS: Endothelial nitric oxide synthase
NT-pro-BNP: N-terminal pro-brain natriuretic peptide
PTH: Parathyroid hormone
QC: Quality control
RTR: Renal transplant recipients
SBP: Systolic blood pressure

## References

[CR1] Abedini S, Meinitzer A, Holme I (2010). Asymmetrical dimethylarginine is associated with renal and cardiovascular outcomes and all-cause mortality in renal transplant recipients. Kidney Int.

[CR2] Albrecht EW, Stegeman CA, Tiebosch AT (2002). Expression of inducible and endothelial nitric oxide synthases, formation of peroxynitrite and reactive oxygen species in human chronic renal transplant failure. Am J Transplant.

[CR3] Baia LC, Van den Berg E, Vervloet MG (2014). Fish and omega-3 fatty acid intake in relation to circulating fibroblast growth factor 23 levels in renal transplant recipients. Nutr Metab Cardiovasc Dis.

[CR4] Boger RH, Bode-Boger SM, Szuba A (1998). Asymmetric dimethylarginine (ADMA): a novel risk factor for endothelial dysfunction: its role in hypercholesterolemia. Circulation.

[CR5] Can A, Bekpinar S, Gurdol F (2011). Dimethylarginines in patients with type 2 diabetes mellitus: relation with the glycaemic control. Diabetes Res Clin Pract.

[CR6] Cooke JP (2000). Does ADMA cause endothelial dysfunction?. Arterioscler Thromb Vasc Biol.

[CR7] Davids M, Teerlink T (2013). Plasma concentration of arginine and asymmetric dimethylarginine do not reflect their intracellular concentrations in peripheral blood mononuclear cells. Metabolism.

[CR8] Davids M, van Hell AJ, Visser M (2012). Role of the human erythrocyte in generation and storage of asymmetric dimethylarginine. Am J Physiol Heart Circ Physiol.

[CR9] Dubois D, Dubois EF (1989). A formula to estimate the approxiamte surface area if height and wight be known 1916. Nutrition.

[CR10] Duckelmann C, Mittermayer F, Haider DG (2007). Asymmetric dimethylarginine enhances cardiovascular risk prediction in patients with chronic heart failure. Arterioscler Thromb Vasc Biol.

[CR11] Faraci FM, Brian JE, Heistad DD (1995). Response of cerebral blood vessels to an endogenous inhibitor of nitric oxide synthase. Am J Physiol.

[CR12] Fliser D, Kronenberg F, Kielstein JT (2005). Asymmetric dimethylarginine and progression of chronic kidney disease: the mild to moderate kidney disease study. J Am Soc Nephrol.

[CR13] Gardiner SM, Kemp PA, Bennett T (1993). Regional and cardiac haemodynamic effects of NG, NG, dimethyl-l-arginine and their reversibility by vasodilators in conscious rats. Br J Pharmacol.

[CR14] Hanai K, Babazono T, Nyumura I (2009). Asymmetric dimethylarginine is closely associated with the development and progression of nephropathy in patients with type 2 diabetes. Nephrol Dial Transplant.

[CR15] Hariharan S, McBride MA, Cherikh WS (2002). Post-transplant renal function in the first year predicts long-term kidney transplant survival. Kidney Int.

[CR16] Huang PL, Huang Z, Mashimo H (1995). Hypertension in mice lacking the gene for endothelial nitric oxide synthase. Nature.

[CR17] Kielstein JT, Impraim B, Simmel S (2004). Cardiovascular effects of systemic nitric oxide synthase inhibition with asymmetrical dimethylarginine in humans. Circulation.

[CR18] Leiper JM (2005). The DDAH-ADMA-NOS pathway. Ther Drug Monit.

[CR19] Levey AS, Stevens LA, Schmid CH (2009). A new equation to estimate glomerular filtration rate. Ann Intern Med.

[CR20] Lu TM, Chung MY, Lin CC (2011). Asymmetric dimethylarginine and clinical outcomes in chronic kidney disease. Clin J Am Soc Nephrol.

[CR21] Malyszko J, Koc-Zorawska E, Matuszkiewicz-Rowinska J, Malyszko J (2014). FGF23 and Klotho in relation to markers of endothelial dysfunction in kidney transplant recipients. Transplant Proc.

[CR22] Meinitzer A, Kielstein JT, Pilz S (2011). Symmetrical and asymmetrical dimethylarginine as predictors for mortality in patients referred for coronary angiography: the Ludwigshafen Risk an Cardiovascular Health study. Clin Chem.

[CR23] Mihout F, Shweke N, Bige N (2011). Asymmetric dimethylarginine (ADMA) induces chronic kidney disease through a mechanism involving collagen and TGF-beta1 synthesis. J Pathol.

[CR24] Nakayama T, Sato W, Kosugi T (2009). Endothelial injury due to eNOS deficiency accelerates the progression of chronic renal disease in the mouse. Am J Physiol Renal Physiol.

[CR25] Ravani P, Tripepi G, Malberti F (2005). Asymmetrical dimethylarginine predicts progression to dialysis and death in patients with chronic kidney disease: a competing risks modeling approach. J Am Soc Nephrol.

[CR26] Shi B, Ni Z, Zhou W (2010). Circulating levels of asymmetric dimethylarginine are an independent risk factor for left ventricular hypertrophy and predict cardiovascular events in pre-dialysis patients with chronic kidney disease. Eur J Intern Med.

[CR27] Surdacki A, Nowicki M, Sandmann J (1999). Reduced urinary excretion of nitric oxide metabolites and increased plasma levels of asymmetric dimethylarginine in men with essential hypertension. J Cardiovasc Pharmacol.

[CR28] Tripepi G, Kollerits B, Leonardis D et al (2015) Competitive interaction between fibroblast growth factor 23 and asymmetric dimethylarginine in patients with CKD. J Am Soc Nephrol 26:935–944 10.1681/ASN.2013121355 PMC4378102 25150156

[CR29] Tsikas D, Schubert B, Gutzki FM (2003). Quantitative determination of circulating and urinary asymmetric dimethylarginine (ADMA) in humans by gas chromatography-tandem mass spectrometry as methyl ester tri(N-pentafluoropropionyl) derivative. J Chromatogr B Analyt Technol Biomed Life Sci.

[CR30] van den Berg E, Engberink MF, Brink EJ (2012). Dietary acid load and metabolic acidosis in renal transplant recipients. Clin J Am Soc Nephrol.

[CR31] van den Berg E, Geleijnse JM, Brink EJ (2012). Sodium intake and blood pressure in renal transplant recipients. Nephrol Dial Transplant.

[CR32] van den Berg E, Pasch A, Westendorp WH (2014). Urinary sulfur metabolites associate with a favorable cardiovascular risk profile and survival benefit in renal transplant recipients. J Am Soc Nephrol.

[CR33] Vogelzang JL, van Stralen KJ, Noordzij M et al (2015) Mortality from infections and malignancies in patients treated with renal replacement therapy: data from the ERA-EDTA registry. Nephrol Dial Transplant 30:1028–1037 10.1093/ndt/gfv007 25637641

[CR34] Yilmaz MI, Saglam M, Caglar K (2006). The determinants of endothelial dysfunction in CKD: oxidative stress and asymmetric dimethylarginine. Am J Kidney Dis.

[CR35] Yilmaz MI, Sonmez A, Saglam M (2010). FGF-23 and vascular dysfunction in patients with stage 3 and 4 chronic kidney disease. Kidney Int.

[CR36] Zoccali C, Mallamaci F, Maas R (2002). Left ventricular hypertrophy, cardiac remodeling and asymmetric dimethylarginine (ADMA) in hemodialysis patients. Kidney Int.

